# Cytochromes *c* in Archaea: distribution, maturation, cell architecture, and the special case of *Ignicoccus hospitalis*

**DOI:** 10.3389/fmicb.2015.00439

**Published:** 2015-05-12

**Authors:** Arnulf Kletzin, Thomas Heimerl, Jennifer Flechsler, Laura van Niftrik, Reinhard Rachel, Andreas Klingl

**Affiliations:** ^1^Department of Biology, Sulfur Biochemistry and Microbial Bioenergetics, Technische Universität DarmstadtDarmstadt, Germany; ^2^Fakultät für Biologie und Vorklinische Medizin, Zentrum für Elektronenmikroskopie, Universität RegensburgRegensburg, Germany; ^3^Department of Microbiology, Institute for Water and Wetland Research, Radboud University NijmegenNijmegen, Netherlands; ^4^Department of Biology I, Plant Development, Biocenter LMU MunichPlanegg-Martinsried, Germany

**Keywords:** cytochrome *c*, Archaea, *Ignicoccus hospitalis*, ANME, anammox planctomycetes, bioinformatics, molecular modeling

## Abstract

Cytochromes *c* (Cytc) are widespread electron transfer proteins and important enzymes in the global nitrogen and sulfur cycles. The distribution of Cytc in more than 300 archaeal proteomes deduced from sequence was analyzed with computational methods including pattern and similarity searches, secondary and tertiary structure prediction. Two hundred and fifty-eight predicted Cytc (with single, double, or multiple heme *c* attachment sites) were found in some but not all species of the *Desulfurococcales, Thermoproteales, Archaeoglobales, Methanosarcinales, Halobacteriales*, and in two single-cell genome sequences of the *Thermoplasmatales*, all of them *Cren*- or *Euryarchaeota*. Other archaeal phyla including the *Thaumarchaeota* are so far free of these proteins. The archaeal Cytc sequences were bundled into 54 clusters of mutual similarity, some of which were specific for Archaea while others had homologs in the Bacteria. The cytochrome *c* maturation system I (CCM) was the only one found. The highest number and variability of Cytc were present in those species with known or predicted metal oxidation and/or reduction capabilities. Paradoxical findings were made in the haloarchaea: several Cytc had been purified biochemically but corresponding proteins were not found in the proteomes. The results are discussed with emphasis on cell morphologies and envelopes and especially for double-membraned Archaea-like *Ignicoccus hospitalis*. A comparison is made with compartmentalized bacteria such as the *Planctomycetes* of the Anammox group with a focus on the putative localization and roles of the Cytc and other electron transport proteins.

## Introduction

**Figure 1:**
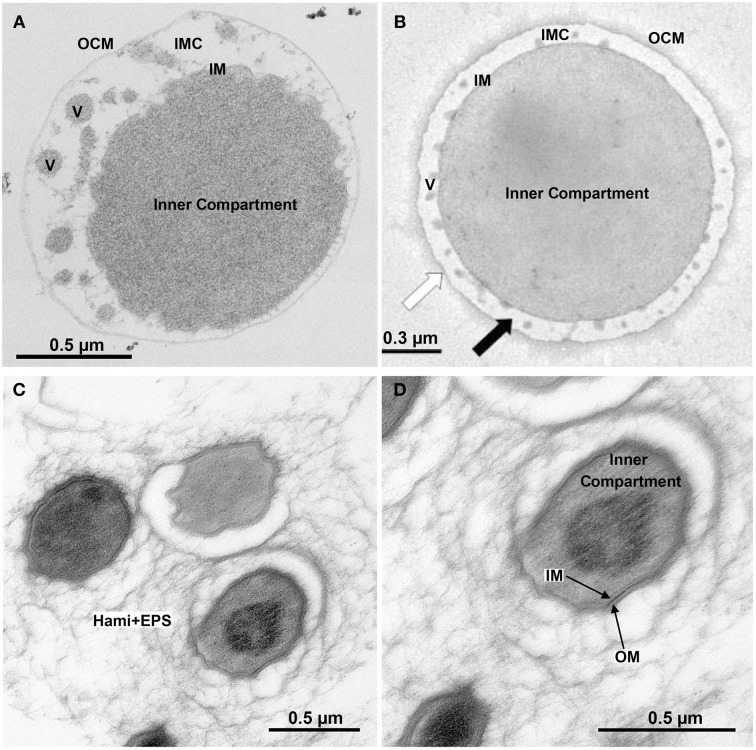
**Ultrastructure of double-membraned Archaea**. Transmission electron micrographs of ultrathin sections of high-pressure frozen, Epon-embedded cells; **(A)** *Ignicoccus hospitalis*; **(B)** *Methanomassiliicoccus luminyensis* taken and modified from Dridi et al. (2012), with permission from the *International Journal of Systematic and Evolutionary Microbiology*, confirmation number 11261287; **(C,D)** *Candidatus* “Altiarchaeum hamiconexum.” Abbreviations and explanation: IMC, intermembrane compartment; IM, inner membrane; OCM, outer cellular membrane; V, vesicles; EPS, extracellular polymeric substances; Hami, extracellular long hooked pili (Moissl et al., 2005).

The chemolithotrophic, hyperthermophilic Archaeon *Ignicoccus hospitalis* is unusual in several aspects (Huber et al., 2012). First, it is the only host of the symbiotic and/or parasitic Archaeon *Nanoarchaeum equitans*. Second, *I*. *hospitalis* cells do not possess a cell wall. Instead they comprise two membrane systems: an inner membrane (IM) encompassing the densely contrasted inner compartment, which contains DNA, ribosomes, and presumably many biosynthetic enzymes (Figure 1; Huber et al., 2012). The outer cellular membrane (OCM) surrounds the cell and contains regularly arrayed small hydrophobic proteins (Burghardt et al., 2007; Huber et al., 2012). A lightly contrasted intermembrane compartment separates both membranes (IMC, 50–1000 nm in width). The IMC contains densely contrasted tubes and vesicles directly involved in the interplay between both membranes (Huber et al., 2012; Meyer et al., 2014). The energy-converting enzymes ATP synthase, hydrogenase, sulfur reductase, and acetyl-CoA synthase are located in the OCM representing the cellular and bioenergetic boundary of the cell from the non-living environment (Küper et al., 2010; Mayer et al., 2012). Therefore, the OCM of *I*. *hospitalis* is not equivalent to the outer membrane of Gram-negative bacteria (Huber et al., 2012).

The coloration of *I*. *hospitalis* cells is a third unusual aspect: soluble extracts and membrane fractions are brightly red resulting from a high content of soluble and membrane-bound cytochromes *c* (Cytc). We had purified three different Cytc from *I*. *hospitalis* cells, however, we can so far only speculate about their *in vivo* function (Naß et al., 2014). Two of these proteins, named Igni_0955 and Igni_1359 after their GenBank locus tag numbers, were present in soluble and membrane extracts, while the third one (Igni_0530) was present only in the membrane fractions.

Cytochromes *c* are widely distributed in the living world. For example, *Pseudomonas, Paracoccus*, and *Thermus* species possess the genes for the canonical mitochondrial-type respiratory chain including the *bc*_1_ complex (complex III) and the soluble monoheme Cytc as electron carrier between complexes III and IV (reviewed, for example, in Mooser et al., 2006; Noor and Soulimane, 2013). Among Archaea, the bioenergetics and the composition of electron transport chains was most thoroughly studied in *Pyrococcus/Thermococcus* spp., methanogens, haloarchaea, and *Sulfolobales* (Schäfer et al., 1999; Schäfer, 2004; Thauer et al., 2008; Mayer and Müller, 2014). Among these, only those methanogens of the *Methanosarcinales* order possess Cytc, whereas they were not detectable—biochemically or by sequence comparisons—in the other taxa or in other methanogens (Thauer et al., 2008).

The hallmark of Cytc is a covalent ligation of a heme *b* moiety to the protein backbone. In most cases, two cysteine side chains—usually present in a sequence motif CxxCH—form thioether linkages to the heme backbone. The histidine provides the proximal axial ligand of the octahedral coordination sphere of the iron in the center of the heme. The distal axial ligand comes from a distant His, Met or, less frequently, other residues. Variations of this theme may involve penta- instead of hexa-coordinated hemes as for example in Cytc', a CxxCK heme-binding motif (e.g., in nitrite reductases; Lockwood et al., 2011) or different spacing of the cysteine residues (Kern et al., 2011). Motif variations usually occur in multiheme cytochromes *c* (MCC) acting as enzymes and not as electron transfer proteins.

The double thioether linkage is formed by maturation proteins, which are grouped by phylogenetic and functional relationship into five systems (Allen et al., 2006; Allen, 2011; de Vitry, 2011; Simon and Hederstedt, 2011; Stevens et al., 2011). In most bacteria, Cytc maturation (CCM) takes place on the positive (p) side of the cytoplasmic membrane (maturation systems I and II; Simon and Hederstedt, 2011; Stevens et al., 2011). The apoproteins are transported across the cytoplasmic membrane by the General Secretory Pathway (GSP) (Sec-System) so that they carry a recognizable signal sequence at their N-termini, which is—apart from the CxxCH motif—the second feature important for bioinformatic prediction of these proteins. System I or CCM (Cytc maturation) consists of up to nine different proteins including a heme ligase, chaperones, ATP-transporters, and protein disulfide isomerases (Stevens et al., 2011; Verissimo and Daldal, 2014). It occurs in Alpha- and other Gammaproteobacteria and it was identified in Archaea during a previous study (Allen et al., 2006). System II consists of less and mostly unrelated proteins compared to System I (Simon and Hederstedt, 2011).

The number of studies conducted about occurrence and function of Cytc in Archaea is limited and no systematic survey was so far performed. Apart from *I*. *hospitalis* (Naß et al., 2014), Cytc were found biochemically in the hydrogen-oxidizing and sulfur-reducing complex of the related Archaea *Pyrodictium abyssi* and *P*. *brockii* (Pihl et al., 1992; Dirmeier et al., 1998), in a *bc_1_* complex from the likewise related microaerophilic *Aeropyrum pernix* (Kabashima and Sakamoto, 2011), in the nitrate reducer *Pyrobaculum aerophilum* (Feinberg and Holden, 2006; all of them hyperthermophilic *Crenarchaeota*), in cultured (*Methanosarcina* spp.) and uncultured species (ANME-1 and ANME-2) of the *Methanosarcinales* and in several haloarchaea (all Euryarchaeota; Kamlage and Blaut, 1992; Scharf et al., 1997; Sreeramulu et al., 1998; Sreeramulu, 2003; Meyerdierks et al., 2010; Wang et al., 2011, 2014). Surprisingly, experimental gene identification was accomplished only for a few of these species including the three multiheme Cytc from *I. hospitalis* and of the *bc_1_* complex of the related *A*. *pernix* (Kabashima and Sakamoto, 2011; Naß et al., 2014).

When looking at *I*. *hospitalis* and trying to put the pieces of this puzzle together, questions arise about the distribution of Cytc in different types of archaeal cells, about their targeting and about the nature and location of the biogenesis system. Since occurrence and distribution of Cytc in Archaea was not recently analyzed in detail, we present here the results of a systematic computational survey. The results are discussed with respect to cell ultrastructure and the physiology of the different archaeal with a special focus on the comparison of *I. hospitalis* with other single, double, and triple-membraned Archaea and Bacteria.

## Materials and methods

### Bioinformatic procedures

The complete non-redundant set of archaeal proteins was downloaded July 23rd, 2014 from Uniprot database in FASTA format (http://www.uniprot.org/). In addition, archaeal sequences deposited at GenBank in 2014 were downloaded Janurary 6th, 2015, from the non-redundant protein database (NR). Both sets of sequences were curated for duplicate species and combined. The total set of 883,607 proteins (Table 1) were analyzed in installments of up to 30,000 sequences for the amino acid pattern CxxCH using the 3of5 algorithm (Seiler et al., 2006) installed locally at the HUSAR Sequence Analysis Facility at the German Cancer Research Center, Heidelberg (http://genius.embnet.dkfz-heidelberg.de/menu/w2h/w2hdkfz/; 3of5 web server available at http://www.dkfz.de/mga2/3of5/3of5.html). The hits (Table 1) were converted into a tab-delimited list of accession numbers and corresponding hit motifs using the advanced “find and replace” features of Microsoft Word and finally inserted into a Microsoft Excel work sheet (Table S1). A list of database accession numbers (Uniprot identifiers and GenBank GI numbers) was generated from the appropriate Excel column and the full FASTA-formatted sequences were retrieved from the respective databases. They were also converted into a tab-delimited format and incorporated into the Excel table. Delimiters (§, $, #) were placed into additional columns for re-formatting purposes. For addition of the locus tags, the same set of sequences was retrieved in GenBank format, reformatted as above and copied into a separate work sheet. The column with the locus tags or gene designations was copied into the main table as appropriate.

**Table 1:** **Statistics of cytochrome c prediction in Archaea**.

Total No. of archaeal proteins	888,023
Uniprot non-redundant proteins July 2014	816,158
Genbank additional archaeal proteins	71,865
No. of defined species/genomes strains	312
Total hits with CxxCH search	4795
No. of duplicated sequence hits	563
No. of unique sequences among duplicates	222
Multiheme cytochrome *c* candidate (No_CxxCH_ ≥ 3 per sequence)	179
Same with N-term.TMH and/or predicted signal seq.	159
**No. of predicted multiheme cytochromes *c***	**167**
No. of species/strains (cultured or uncultured) with multiheme cytochromes *c*	29
False positives (e.g., RecJ; 3× CxxCH each)	12
No. of proteins with 2 CxxCH/sequence	206
Same with N-term.TMH and/or predicted signal/TAT seq.	24
**No. of predicted diheme cytochromes *c***	**28**
No. of species (cultured or uncultured) with diheme cytochromes *c*	20
False positives	178
No. of proteins with 1 CxxCH/sequence	4410
Same with N-term.TMH and/or predicted signal seq.	157
**No. of predicted monoheme cytochromes *c* candidates**	**64**
No. of species (cultured or uncultured) with monoheme cytochromes *c*	39
False positives	4347
Total No. of proteins subjected to structure prediction	1754
No. of cytochrome *c* candidates clustered for detailed analysis	350
**No. of predicted archaeal cytochrome *c* proteins**	**258**
No. of sequence similarity clusters	54
No. of predicted archaeal Cytc in species with 3 or more *ccm* genes^a^	241
No. of species/environmental samples	47
No. of predicted archaeal Cytc in species with 0–1 *ccm* genes^b^	17
No. of species	17

a See **Figure 2a**;

b *See **Figure 2b***.

The set of 4795 hit sequences was analyzed for transmembrane helices (TMH) using the TMHMM (one line per protein; http://www.cbs.dtu.dk/services/TMHMM-2.0/; Krogh et al., 2001) and SOSUI batch servers (http://harrier.nagahama-i-bio.ac.jp/sosui/; Hirokawa et al., 1998). The results were reformatted and again copied to the main table (Table S1). Signal sequences were predicted using SignalP (http://www.cbs.dtu.dk/services/SignalP/, model for Gram-negative bacteria; Petersen et al., 2011) and TatP (http://www.cbs.dtu.dk/services/TatP/; Bendtsen et al., 2005) for GSP and twin-arginine protein translocation (TAT) signal peptides, respectively. Proteins were also analyzed using OCTOPUS in cases of manually identified Cytc candidates with no result in the N-terminal TMH prediction. Sequences with three or more CxxCH motifs were defined as multiheme Cytc (MCC) unless shown not to be—by a high similarity to known non-cytochrome proteins in BLASTP searches (e.g., RecJ homologs). Additionally, various known Cytc and MCCs were used to query the Archaea subsection of the GenBank protein database. Sequences with two or one CxxCH motif were considered Cytc candidates if they contained an N-terminal TMH and/or a signal sequence. Candidates were subjected to three-dimensional modeling using the batch processing mode of the Phyre^2^ server (http://www.sbg.bio.ic.ac.uk/phyre2/html/page.cgi?id=index; Kelley and Sternberg, 2009). The results were purged from non-significant models (i.e., low confidence and/or alignment coverage percentage) and significant hits were used to evaluate the previously defined Cytc candidate clusters for completeness and correct identification. The *I. hospitalis* Cytc were also modeled using the I-Tasser server with omission of the respective signal sequences (http://zhanglab.ccmb.med.umich.edu/I-TASSER/; Roy et al., 2010). The resulting Igni_0759 model was further adjusted by taking the predicted heme ligand out of the I-Tasser results files. The pdb coordinates including the heme were imported into UCSF Chimera (Pettersen et al., 2004) and the heme position was adjusted manually in order to build the thioether bonds between the heme and the two cysteine side chains followed by energy minimization. In the next round, a bond between the heme iron and the Nε atom of the proximal ligand His_32_ was created and the energy minimization step repeated. The figure was prepared in Pymol (Delano, 2002).

The set of 4795 primary hit sequences was converted into a BLAST database using the standalone BLAST+ program downloaded from NCBI (http://blast.ncbi.nlm.nih.gov/Blast.cgi?CMD=Web&PAGE_TYPE=BlastDocs&DOC_TYPE=Download). Cytc candidates were compared against this database in order to find missing homologs and to identify clusters of mutually similar Cytc candidates. Clusters were aligned separately (Supplementary Alignment File Archaea_Cytc.zip). The multiheme cytochromes identified by Sharma et al. (2010) were also downloaded in FASTA format, converted into a separate BLAST database; they were used for the determination of cluster similarity and to relate clusters defined in this study to those from Sharma et al. (2010; Table S1). The primary hit sequences were also compared using BLASTP against the conserved domain database (CDD) installed locally.

Our methods differed from previous computational studies presented by Bertini et al. (2006) and Sharma et al. (2010). Both used HMMs (Sharma et al. for diheme and multiheme Cytc prediction only) and both used comparison against the protein family database for curation (PFAM; http://pfam.xfam.org/). Many of the cytochromes predicted here are not even clustered in PFAM or in NCBI's CDD for lack of 3D structures and/or biochemical description (Table S1, CDD search) so that we used clustering combined with structure prediction in order to identify Cytc folds in proteins. The main advantages of the methods used here are simplicity and no need for specialized software. They can be repeated from almost any standard PC or Mac using internet-available tools and free software (except for Microsoft Office products). The likewise freely accessible structure prediction part helped in assessing the previous conclusions.

The search for Cytc biosynthesis proteins was performed essentially as described (Allen et al., 2006). For system I, the CcmB, CcmC, CcmE, CcmF proteins from *Methanosarcina acetivorans, A*. *pernix, Haloarcula marismortui* (for GI numbers, see Allen et al., 2006), and *E*. *coli* were used in BLASTP searches against the archaeal proteins. BLASTP searches were repeated with archaeal hit sequences because sequence similarities were often low between unrelated Archaea. The *Leptospira interrogans* CcmH (GI:45656703) was used in addition, as homologs had so far not previously been found in Archaea (Allen et al., 2006). For System II, *Wolinella succinogenes* ResB was used (GI:34484157); for System III, the two heme lyases from *Saccharomyces cerevisiae* were used and for System IV the *Chlamydomonas reinhardtii* CCB1-4 proteins (de Vitry, 2011).

### Electron microscopy

For electron microscopy analysis, fresh *I. hospitalis* cells were cultivated, high-pressure frozen and freeze-substituted in 95% acetone, 0.5% glutaraldehyde, 0.5% uranyl acetate, and 5% water as described (Rachel et al., 2010; Naß et al., 2014). After freeze-substitution fixation, samples were embedded in Epon. For localization of proteins on ultrathin sections, the primary antiserum directed against Igni_0955 was used without further purification. For detection, secondary antibodies coupled to ultra-small gold particles were made visible by silver enhancement. Images were recorded as described (Naß et al., 2014). The *Kuenenia stuttgartiensis* cell had also been high-pressure frozen, freeze-substituted in acetone containing 2% OsO_4_, 0.2% uranyl acetate, and 1% water and Epon-embedded as described (Wu et al., 2012). *Candidatus* “Altiarchaeum hamiconexum” cells were sampled and prepared for electron microscopy as described elsewhere (Perras et al., 2014; Probst et al., 2014a).

## Results

### Prediction of cytochromes *c* and their maturation proteins in Archaea

Motif and similarity searches and homology modeling were applied to the prediction of Cytc and their distribution in Archaea. 4795 archaeal proteins (Table 1) were found to contain at least one CxxCH amino acid pattern (Table S1). One hundred and seventy nine proteins contained at least three CxxCH motifs (defined here as MCCs), among those, 159 had a recognizable signal sequence and/or a predicted transmembrane helix (TMH) at their N-termini (Table 1). 12 sequences with three CxxCH motifs each were identified with BLASTP searches as RecJ exonuclease homologs and were considered as false positives. RecJ family proteins with 1–3 CxxCH motifs were among the most common random hits in the motif searches. The remaining 167 proteins from 29 archaeal species/strains were considered as multiheme cytochromes *c* (MCC; Table 1 and Table S1).

The prediction of di- and mono-heme Cytc from the motif search resulted in a higher proportion of non-specific hits. Twenty eight out of 206 proteins from 20 species were identified as diheme Cytc candidates (Tables S1, S2). The majority of 4410 proteins with a single CxxCH motif (Table 1) were random hits with no recognizable similarity to Cytc or any feature suggestive of them being one. Among the 229 proteins with an N-terminal TMH and/or signal sequence, only those were considered as Cytc candidates if they were either similar to known Cytc sequences (e.g., cluster 30, homologs of the *A*. *pernix bc_1_* complex), or if the CxxCH motif was conserved in a significant percentage of the homologs found in BLAST searches, and if the proteins were not *bona fide* members of other known protein families. Thioredoxin family proteins (including protein disulfide isomerases) were frequently occurring false positives with an N-terminal TMH; subunits of RNA and DNA polymerases, molybdopterin biosynthesis proteins, endonucleases, Zn^2+^-binding domains, and iron-sulfur proteins were among the most frequent false positives without a TMH.

One thousand seven hundred and fifty four proteins annotated as “hypotheticals” were subjected to batch structure prediction. The fold recognition often gave necessary hints for the decision whether a protein or a cluster represents Cytc. No further Cytc candidates were spotted in this subset of the data. After reducing the score to 154 monoheme Cytc candidates falling into 30 similarity clusters (Tables S1, S2), 3D structure prediction was performed showing that 9 clusters all gave ≥ 96% confidence predictions with various Cytc, the prediction results of cluster 47 were considered of intermediate quality (90% confidence). This and cluster 38 were included in the Cytc group. Seventeen sequence clusters were excluded from the Cytc group mostly because they gave significant modeling results with known non-Cytc proteins.

#### Multiheme cytochromes *c* in Archaea

With one exception (Figure 2), the presence of MCCs-encoding genes was restricted to four of the major archaeal orders: the *Desulfurococcales*, the genus *Pyrobaculum* within the order of the *Thermoproteales* (both *Crenarchaeota*), the *Archaeoglobales*, and the *Methanosarcinales* including the methane-oxidizing environmental candidate species of the ANME-1 and ANME-2 groups (Figures 2, 3). The highest numbers of predicted MCC were found encoded in those species known or suspected to thrive anaerobically by iron respiration like *F*. *placidus* and in the uncultured methane-oxidizing Archaea of the ANME-1 and ANME-2 groups. The maximal number of CxxCH motifs in a single sequence was 33 in a large protein from the euryarchaeote *Ferroglobus placidus* (Figure 2).

**Figure 2:**
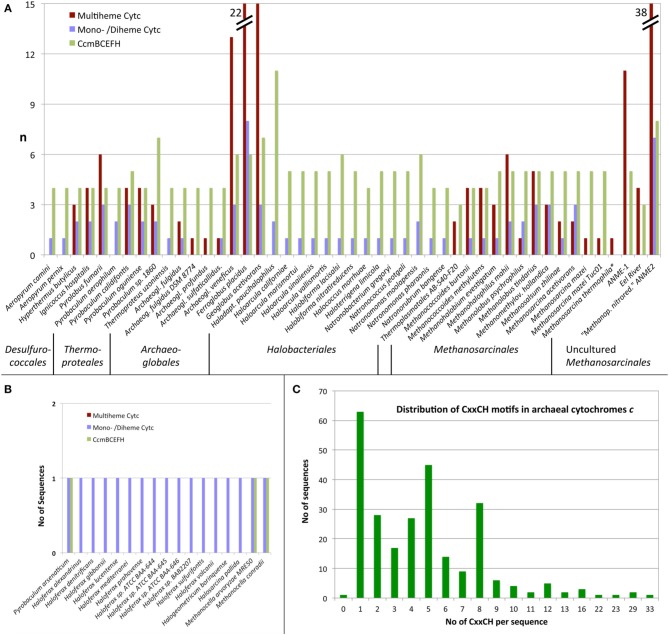
**Distribution of cytochromes *c* in Archaea predicted from protein sequences. (A,B)** Number of predicted MCCs, combined diheme and monoheme Cytc and number of CcmB, CcmC, CcmE, CcmF, and CcmH homologs per proteome in species with **(A)** and without **(B)** significant number (≥3) of CCM homologs; **(C)** Frequency of CxxCH motifs per protein.

**Figure 3:**
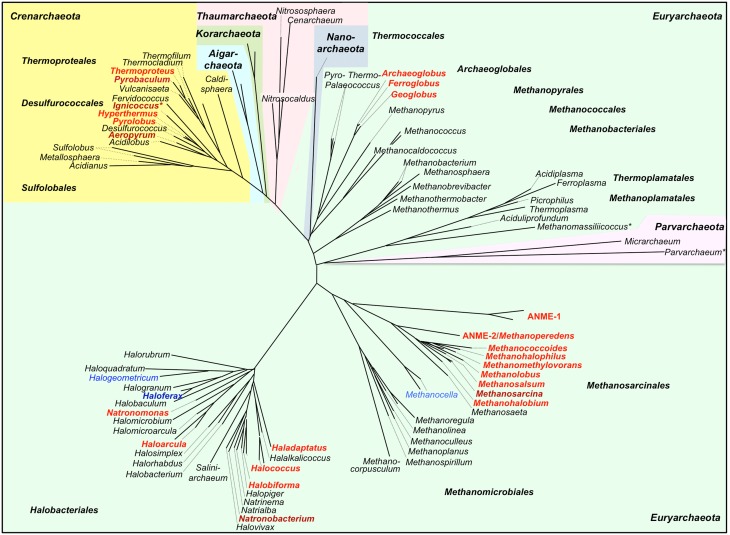
**Phylogenetic 16S rDNA dendrogram of the Archaea and distribution of predicted cytochrome *c* genes**. The dendrogram was made from a 16S alignment both calculated with MAFFT (Katoh and Standley, 2014; http://mafft.cbrc.jp/alignment/server/). Dark red/dark blue, Cytc biochemically found; red, Cytc-encoding and *ccm* genes found (Figure 2A); blue, Cytc candidate genes but no or single *ccm* genes found (Figure 2B); *Archaea with double membranes. Note: This dendrogram was created to depict the distribution of Cytc in Archaea and is based on 16S sequences only. The branching order of archaeal phyla does not correlate with more advanced dendrograms based on concatenated proteins sets. For a discussion of large-scale archaeal phylogeny, see for example Guy and Ettema (2011); Forterre (2013), and Petitjean et al. (2014).

The predicted MCCs were grouped in 34 clusters according to sequence similarity (Tables S1, S2, multiple alignments in the compressed supplemental sequence file Archaea_Cytc.zip). Some of the archaeal MCCs belong to well-known families like the hydroxylamine oxidoreductases (sequence cluster No. 4; 11 hits), octaheme tetrathionate reductases (cluster No. 5; 7 hits), or the periplasmic nitrite reductases (No. 69; 3 hits). In contrast, the protein function of most of the MCCs from Archaea is not known; many do not even have bacterial counterparts (e.g., clusters 1, 2, 11, 12 etc.; Table S2). Sometimes, structure prediction of MCC candidate proteins gave high-confidence (100%), full-length predictions. For example, protein models of cluster 1 matched with *Thioalkalivibrio nitratireducens* octaheme nitrite reductase (PDB accession 3f29) despite undetectably low sequence similarity, so that their function might nevertheless be inferred. Proteins of cluster 2 matched structurally octaheme tetrathionate reductases (PDB 1sp3; cluster 5). Other clusters gave more ambiguous results, which must be handled with care (Table S2), especially, when the number of CxxCH motifs in models and templates differed (e.g., clusters 8 and 9; not shown).

#### Di- and mono-heme cytochromes *c*

Among the predicted diheme Cytc (seven similarity clusters; Table S2 and (Supplementary File Archaea_Cytc.zip) were peroxidases of the MauG type (cluster 29), thiosulfate dehydrogenases (TDH; cluster 28), a *bc_1_* complex homolog from hyperthermophile *Pyrolobus fumarii* (cluster 30; the homologs from three other *Desulfurococcales* species have only one CxxCH motif including the biochemically characterized APE_1719 from *A. pernix*; Kabashima and Sakamoto, 2011), and Split-Soret Cytc (cluster 21), the latter with predicted Twin-Arginine signal peptides. Six of these proteins were from *Archaeoglobales*, two from the crenarchaeote *Pyrolobus fumarii*, four from *Methanosarcinales* while the remaining 16 proteins were from various haloarchaea, which do not harbor MCCs as far as we know (Figure 2).

Structure prediction of the MauG peroxidases, the *bc_1_* complex homologs and the Split-Soret Cytc were consistent with the templates and they covered ≥ 70% of the respective proteins with 100% confidence (Table S2). More interesting was the case of the TDH homologs (cluster 28): modeling suggested structural similarity to SoxA proteins, which catalyze, together with SoxX, the oxidative transfer of thiosulfate to a cysteine side chain of SoxYZ. Sequence similarity between these two sulfur cycle enzymes is low but modeling showed structural similarity. These archaeal TDH homologs are encoded in genomes of five haloarchaeal species in operon-like arrangements with genes for CCM proteins.

Sharma et al. (2010) had predicted MCCs (with 2 hemes/per protein or more instead of at least 3 hemes used here) in 8 out of 47 then-available archaeal genome sequences. We found all of those with the methods used in this study, however our interpretation was sometimes different. For example, they had identified *Methanospirillum hungatei* Mhun_1396 and its paralog Mhun_1882 as putative diheme Cytc. The proteins are highly conserved in methanogens but the CxxCH motifs are not so that we disregarded these two candidates. We also identified many previously unrecognized MCCs annotated as hypotheticals in genome sequences.

Sixty four proteins were assigned as monoheme Cytc candidates from 12 sequence clusters (Table 1 and Table S1). The modeling approach gave results with templates like cytochrome *c*_(2)_, cytochrome P460, SoxX, and Cytc subunits of NO reductase (NorC) or ethylbenzene dehydrogenase (Table S2). A special case is the nitrite reductase subunit Pars_0592 from *Pyrobaculum arsenaticum*, which was identified with BLASTP searches and which is similar to its heme-*c* containing homologs (68 and 52% identity to the two *P. aerophilum* proteins PAE3598 and PAE1347, respectively) but which has a tyrosine residue instead of the first cysteine in the classical CxxCH motif. We suspect that there might be single or no covalent heme ligation in an otherwise functional protein.

#### Cytochrome *c* maturation proteins

Cytochromes *c* require maturation by heme ligases and, in most cases, transport proteins for the transfer of the heme moiety across the membrane to the electrochemically positive side. Cytochrome *c* maturation system I (CCM) originally described from *E. coli* is one the two most common and the most complex CCM machinery of five known systems. The search for CCM proteins encoded in archaeal genomes was mainly done with sequence comparisons using BLAST and the CcmB, C, E, F, and H proteins as described by Allen et al. (2006) and in the Materials and Methods Section. The *I. hospitalis* genome encodes four proteins, CcmB, CcmC, CcmE, and CcmF indicative of the presence of the entire CCM system. CcmH homologs were solely found in *Ferroglobus placidus*, while the remaining four proteins had homologs in 45 archaeal species, in which Cytc proteins were also predicted (Figure 2). Proteins of cytochrome maturation systems II–V were not identified in Archaea. It can be concluded from these results that the Cytc apoproteins are transferred at least over one membrane. Seventeen monoheme Cytc were predicted in species with either none (*Haloferax spp., Halogeometricum borinquense, Halosarcina pallida*, cluster 50) or only one maturation protein (*Methanocella* spp. *Pyrobaculum arsenaticum*) encoded in the genomes (Figure 2).

### Cytochromes *c* in *Ignicoccus hospitalis*

We had previously reported on the purification of three multiheme cytochromes *c* (MCC) from the hyperthermophilic archaeon *I. hospitalis* (Naß et al., 2014). We had also reported that one of those cytochromes was a membrane-bound MCC with four CxxCH motifs (locus tag Igni_0530) and that two octaheme MCCs were present both in the soluble and the membrane fractions (Igni_1359 and Igni_0955). We had further predicted an octaheme tetrathionate reductase-like protein (Igni_1130) and two so far hypothetical monoheme cytochromes *c* in the *I. hospitalis* proteome (Igni_0579 and Igni_1052; cluster 38). Here, we wanted to investigate in more detail whether the structure prediction used in Cytc identification in Archaea could substantiate this claim. We also extended structure prediction to the MCCs, again with the scope of extending the method more generally.

**Figure 4:**
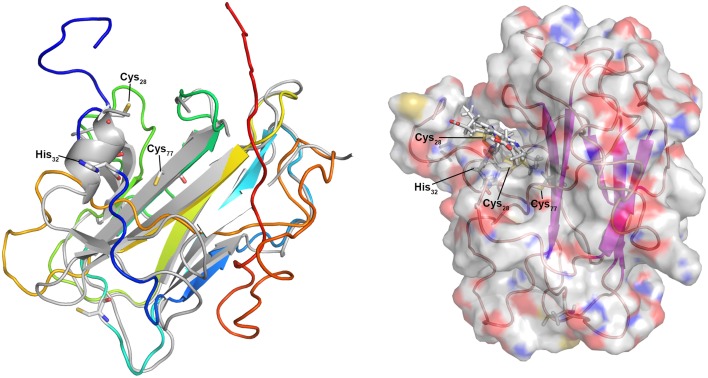
**3D model of the predicted monoheme cytochrome *c* Igni_0759 from *I. hospitalis*. Left panel:** 3D model created using the I-Tasser server (Roy et al., 2010; rainbow color, N-terminus blue) and superimposed over the modeling template (heparin-binding reelin-N domain of f-spondin; PDB accession 3COO; Tan et al., 2008); **Right panel:** Igni_0759 3D model with heme *c* connected to Cys_28_ and Cys_31_ side chains with the iron connected to His_32_.

Igni_0579 and Igni_1052 are similar; Igni_1052 however has a second predicted TMH at its C-terminus not present in Igni_0759. Homologs occur in the related crenarchaeota *Pyrolobus fumarii* and *Hyperthermus butylicus*, both with a C-terminal TMH. The modeling servers (Phyre^2^ and I-Tasser) both used eukaryal spondin as the folding template (a non-heme protein, Tan et al., 2008) with high statistical confidence (100%). The models left a cleft in the molecule sufficient for heme accommodation with the cysteine side chains positioned at the top of the cleft (Figure 4), thus pointing to a space where the heme might be positioned. In further modeling steps, the heme moiety was added to the Igni_0759 model PDB coordinate file and connected to the side chains of Cys_28_ and Cys_31_. After energy minimization, the iron atom was connected to His_32_ as proximal ligand and the protein was again subjected to energy minimization resulting in the model depicted in Figure 4. A further step connecting the iron to the side chain of Cys_77_ as putative distal ligand failed. His_111_ is a second candidate for the distal ligand and it is conserved in the homologs (cluster38_Igni_0759.fasta in the Archaea_Cytc.zip file). It was located beneath the β-sandwich forming the main structural body of the model so that we cannot presently decide, which of these two is correct. In summary, the model is congruent with the hypothesis that these *I. hospitalis* proteins are Cytc and they show that 3D structure prediction could be a valuable tool for the identification of unknown proteins, at least when applied to suspected monoheme Cytc.

Structure prediction was more difficult for the MCCs although Igni_1359 and Igni_0955 gave high-confidence (100%) full-length models with the *Nitrosomonas europeae* HAO 3D structure as template (PDB accession 1FGJ) with up to 28% sequence identity (not shown). Likewise, Igni_1130 gave a well-predicted model with the *Shewanella oneidensis* OTR (3SP3; not shown). However, significant 3D models were also created when the three proteins were modeled with non-homologous MCC templates (e.g., Igni_1130 with the HAO template) regardless of sequence similarity. The MCCs seem to be folded into multiple pre-existing 3D structures because high numbers of heme-binding sites predefine the folding of the apoproteins, thereby restricting the predictive capabilities of structure modeling of MCCs. In consequence, a function prediction of MCC is at best difficult when trying to model non-homologous MCCs of unknown function, while monoheme Cytc give more reliable results.

## Discussion

We present here a study for the identification of Cytc and their maturation proteins encoded in archaeal genomes using a computational approach coupled to an extensive manual evaluation of the results. We show that Cytc are not a common property of the majority of Archaea to our current knowledge and that they are not distributed equally, being restricted to 5–6 of the major taxa (Figure 3). In most Bacteria, Cytc are bound to cytoplasmic membranes or located in the periplasm or—in Gram-positives—in the space containing peptidoglycan and teichoic acids outside the cytoplasmic membrane, which is discussed to be equivalent to the periplasm of Gram-negatives (Matias and Beveridge, 2005). This is different in the compartmentalized Bacteria and Archaea. In the following discussion we will focus on two main questions:

1. What can we learn from the results of our computational study and the present state of knowledge about the distribution of Cytc, physiological patterns, and pathways in different archaeal lineages and about the acquisition of the genes during evolution?
2. What can we learn and predict about the localization and maturation of Cytc in Archaea and especially in double-membraned microorganisms like *I. hospitalis*?

### Cytochromes *c* in Archaea

Forty-seven archaeal species or consortia of uncultured microorganisms were found encoding both Cytc and CCM maturation proteins in their genomes while 17 other species harbor hypothetical single Cytc candidates with little evidence for maturation proteins (Table 1, Figure 2). They belong to only five different orders of Archaea with the exception of two proteins from a single-cell genome of a *Thermoplasmatales* species. Some of the archaeal Cytc have numerous homologs in Bacteria (e.g., clusters 3 and 4) while others are specific for Archaea (e.g., cluster 1–2).

There are differences in the distribution within Cytc-containing archaeal orders and even within single genera: The *Archaeoglobales* are the only order, in which all species sequenced so far contain Cytc genes (Figures 2, 3). In contrast, out of 17 genome-sequenced *Thermoproteales* species only *Thermoproteus uzoniensis* and 4–5 of 7 *Pyrobaculum* spp. contain Cytc genes (Figure 2, Table S1). For example, *Pyrobaculum sp*. strain 1860 and *Pb. oguniense* grow by iron and nitrate respiration (Nunoura et al., 2003; Mardanov et al., 2012) and contain several monoheme Cytc and MCCs obviously involved in various electron transport chains. Two heme-stained proteins were observed in gel electrophoresis of cell extracts of *Pyrobaculum aerophilum* (Feinberg et al., 2008). The authors proposed that they are identical to Cytc subunits of a three-subunit *bc*_1_ complex (PAE1347-9) and of a two-subunit NirS-type *cd*_1_ nitrite reductase (PAE3598). We also found both proteins in this study although the apparent molecular mass of the nitrate-induced band did not match the calculated mass of PAE3598 (20 kDa w/o signal peptide). Protein identification was not given so that ORF numbers of the two heme-stained proteins remain tentative.

The only biochemically purified three-subunit crenarchaeal *bc* complex came from the microaerophilic species *A. pernix* (Kabashima and Sakamoto, 2011). In contrast, cyt *bc* complexes are absent in aerobic *Sulfolobales*, which have an analogous cytochrome *ba* electron transport complex instead (Bandeiras et al., 2009). The Cytc subunit was the only one to be identified (Ape_1719.1). The adjacent gene encodes a subunit of a terminal oxidase, whereas the genes for cytochrome *b* and a Rieske protein are close by but not in the same predicted operon (APE_1724.1 and APE_1725.1). Homologs of Ape_1719.1 are present in *Pyrolobus fumarii* and *Hyperthermus butylicus* but none of the cytochrome *b* and the Rieske proteins. It can be concluded that *Pyrobaculum* spp., *Thermoproteus uzoniensis*, and *Aeropyrum* spp. encode canonical *bc* complexes, whereas the homologous Cytc plays a different role in *Pyrolobus* and *Hyperthermus*, it might be part of an unidentified electron transport complex. The distribution pattern is similar in the remaining archaeal orders with Cytc. Some species of the *Methanosarcinales* and *Halobacteriales* encode single or multiple Cytc and the corresponding *ccm* genes but not the majority of either of them.

#### Cytochromes *c*, anaerobic respiration, and ammonia oxidation

An exceptionally high number of Cytc was found in the euryarchaeota *Ferroglobus placidus* (Figure 2) and *Ca*. “Methanoperedens nitroreducens.” *F. placidus* (and also the crenarchaeote *Pyrolobus fumarii)* grow by Fe^2+^ oxidation with nitrate or Fe^3+^ reduction with various organic and inorganic electron donors, whereas *Ca*. “Mp. nitroreducens” grows by anaerobic oxidation of methane with nitrate (Hafenbradl et al., 1996; Anderson et al., 2011; Haroon et al., 2013). Several ANME Archaea however couple anaerobic methane oxidation to iron or manganese reduction (Beal et al., 2009; Wankel et al., 2012) and the diversity of Cytc in these Archaea was noted in the respective metagenome papers (Meyerdierks et al., 2010; Wang et al., 2014). Some of the large multiheme and multidomain proteins from *F. placidus* and *Ca*. “Mp. nitroreducens” (clusters 17 and 65) have 5–8 CxxCH motifs in their N- or C-terminal Cytc domains. Modeling the none-Cytc domains separately, those parts can be folded into chains of successive beta sandwich domains comparable to surface layer proteins (not shown). The results suggest that these proteins might form extracellular conductive structures or pili as in *Shewanella* or *Geobacter*. Here, periplasmic, outer-membrane, or pilus-bound Cytc transfer electrons to and from the cells (reviewed for example in Gorby et al., 2006; Richter et al., 2012; Boesen and Nielsen, 2013; Smith et al., 2015). This might provide a structural and biochemical basis of the metal ion-reducing and the presumed electron-conductive capabilities of the iron-metabolizing Archaea. In a recent study, many heme-stained bands were found SDS gels of extracts of Fe^3+^-grown *F. placidus* cells. The number of bands and of transcripts of Cytc genes differed depending on the solution state of the iron: there were more Cytc proteins and corresponding transcripts in cells grown on solid compared to soluble Fe^3+^ species; in addition there were numerous type IV pili suggesting close attachment of the cells to the substrate and/or electrically conductive pili (Smith et al., 2015). By analogy, the sulfate reducer *Archaeoglobus veneficus* with a total of 16 Cytc genes should also be able to grow by metal respiration (Figure 2). In summary, metal ion respiration seems to be a predominant motif for the presence of high numbers of Cytc genes in archaeal genomes.

Bacterial sulfate reducers are typical sources of a large variety of Cytc (reviewed for example in Romão et al., 2012) and this seems also true for the *Archaeoglobi* but not for sulfate-reducing crenarchaeota (e.g., *Caldivirga maquilensis*), since we did not find any Cytc genes in the latter microorganisms. Besides sulfate respiration, Cytc play important roles in oxidative and reductive pathways of microbial sulfur and nitrogen cycles such as denitrification, nitrate ammonification, thiosulfate oxidation, and anaerobic ammonium oxidation (Anammox; Kartal et al., 2011; Simon et al., 2011; Kappler and Maher, 2013; van Teeseling et al., 2013). Surprisingly, no Cytc were found in *Thaumarchaeota*, which represent a large phylum of Archaea characterized by their involvement in the global N cycle. *Thaumarchaeota* are proposed to be among the most abundant ammonia oxidizers in marine and in terrestrial ecosystems (Offre et al., 2013; Monteiro et al., 2014; Stieglmeier et al., 2014) and they might be implicated in denitrification as well (Jung et al., 2014). It is therefore surprising that the *Thaumarchaeota* seem to be (so far) devoid of Cytc suggesting that other proteins with comparable activities fill in the gap and that they use different catalytic metal sites.

#### Methanogenesis

Other *Methanosarcinales* species beside the ANME group contain Cytc as it was already discovered in the 1980s (Kuhn et al., 1983; Jussofie and Gottschalk, 1986). Two different Cytc were found spectroscopically in membrane fractions of methanol-grown *Methanosarcina mazei* Gö1 cells but the proteins were not purified or identified (Kamlage and Blaut, 1992). We found three monoheme and one multiheme Cytc gene in the *Ms. mazei* Gö1 genome (Table S1) but their assignment to the proteins reported by Kamlage and Blaut (1992) is currently not possible. Similarly, a multiheme Cytc was found to participate in electron transport of *Ms. thermophila* (Wang et al., 2011). In both cases, the Cytc were oxidized upon heterodisulfide addition (CoM-S-S-CoB) to membrane fractions however their precise role in the redox chains is not known. *Methanosarcinales* species are characterized by their utilization of various C_1_ compounds and many can disproportionate acetate for methanogenesis and energy conservation. Now, *Methanosarcinales* are the only phylogenetic branch of methanogenic euryarchaeota (among at least six others) with both *b* and *c*-type cytochromes but their presence does not seem to be a prerequisite for growth on these substrates. Chemiosmotic coupling during methanogenesis from H_2_/CO_2_ is the most probable reason for the observed higher growth yields in methanogens with cytochromes like *Methanosarcina barkeri* compared to those without (Thauer et al., 2008; Wang et al., 2011). The heterodisulfide reductase from *Ms. barkeri* contains a cytochrome *b* subunit (Heiden et al., 1994; Kunkel et al., 1997). We did not find cytochromes *c* in *Ms. barkeri* in our study here so that the cytochrome *b* subunit alone seems to be responsible for the growth yield effect and there is no other indication that Cytc are integral players in this process. Generally, we observed here that only a small fraction of the known *Methanosarcinales* species contains Cytc suggesting a different role for these proteins in energy metabolism.

#### The haloarchaea paradox

Electron transport components from halophilic Archaea (*Halobacteriales*) were studied since the 1960s (Lanyi, 1968; Cheah, 1970). Later, Scharf et al. (1997) characterized a membrane-bound 2-subunit *bc* complex (14 and 18 kDa, respectively) and a soluble 75 kDa Cytc. A single Cytc candidate was identified in our computational analysis: a 453 aa cytochrome *c*_551_ peroxidase (MauG, cluster 29) is encoded in the genome together with *ccm* genes as in several other haloarchaea and *Methanosarcina* species (Tables S1, S2). This could explain the 75-kDa soluble heme-stained protein (Scharf et al., 1997). In contrast, we could not identify candidates for the heme-*c* protein of the *bc* complex. The situation was similar for *Halobacterium salinarum* and *Haloferax volcanii*. In both species, Cytc were either purified (14 kDa protein in *Hbt. salinarum*), and/or spectroscopically characterized combined with heme-stained SDS gels (Sreeramulu et al., 1998; Tanaka et al., 2002; Sreeramulu, 2003). Two small proteins were found encoded in the *Hfx. volcanii* genome with little mutual sequence similarity and each with homologs in the same 12–13 haloarchaeal species (cluster 35 and 36; Figure 2) not including *Hbt. salinarum*. None of these species contain CCM. Both clusters gave low-confidence structure prediction hits (Table S2) so that independent evidence would be necessary for the identification of the Cytc component of the haloarchaeal *bc* complexes. This leads to the conclusion that they might not be found using similarity and/or pattern searches and that they use non-standard amino acid patterns and heme *c* linkage.

There were several other haloarchaeal species with well-recognized and correctly annotated Cytc and *ccm* genes; cluster 28 comprising 368–485 aa proteins with a monoheme domain and the already mentioned cluster 29 (MauG-type peroxidases). The observation that some haloarchaea contain genes for cluster 28 and 29 Cytc only—the latter occurring in some of the *Methanosarcinales* as well—and the lack of MCCs suggests late gene acquisition from bacterial sources by horizontal gene transfer (HGT) as suggested earlier (Nelson-Sathi et al., 2012). A similar mechanism can be concluded for the metal-metabolizing archaeal species and the *Methanosarcinales*. In conclusion, the overall pattern suggests several events of horizontal transfer from Bacteria to Archaea as proposed as a general model of archaeal gene acquisition (Nelson-Sathi et al., 2015). In addition, the occurrence of Cytc genes seems to match physiological constraints rather than phylogenetic relationship.

### Cytochromes *c* and cell morphology

The majority of Archaea with cytochromes *c*—predicted in this study or biochemically proven—display the “standard” archaeal cell architecture: a cytoplasmic membrane covered with a proteinaceous surface (S-) layer anchored in the membrane (König et al., 2007). S-layers are protein canopies anchored in the cytoplasmic membrane encompassing a “quasi-” or “pseudo-periplasmic space” (Baumeister and Lembcke, 1992; König et al., 2007; Klingl, 2014), which can accommodate membrane-bound and soluble proteins (Baumeister et al., 1989; Veith et al., 2009; Protze et al., 2011; Klingl, 2014). It is therefore to be expected that Cytc are located in this space between cytoplasmic membrane and protein canopy and that they are retained either by pores in the protein lattice or by C-terminal membrane anchors as seen in many of the Cytc candidates described here (Table S1). Similarly, maturation of Cytc should also take place in this environment.

**Figure 5:**
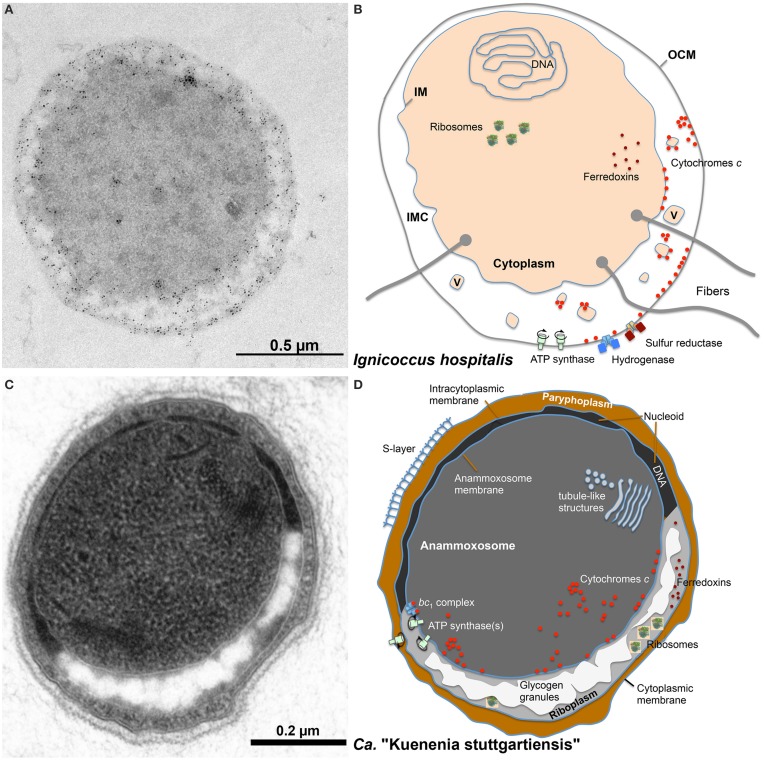
**Comparison of the localization of cytochromes *c* and other important proteins in *I. hospitalis* and in the Anammox bacterium *Candidatus* “Kuenenia stuttgartiensis.”** **(A)** Immuno-labeling of an *I*. *hospitalis* ultrathin section with α-Igni_0955 and gold-labeled α-IgG secondary antibody; **(B)** schematic view of an *I*. *hospitalis* cell with ultrastructural features and known or predicted distribution of proteins/enzymes; **(C)** ultrathin section of *Ca*. “K. stuttgartiensis”; **(D)** schematic view of a *Ca*. “K. stuttgartiensis” cell as above. OCM, outer cellular membrane; IMC, intermembrane compartment; IM, inner membrane; V, vesicles; for the tubule-like structures in the anammoxosome of unknown function see van Niftrik et al. (2008b).

With their two membranes and the lack of an S-layer, *Ignicoccus* species are an exception to the typical archaeal cell architecture (Figures 1, 5). For Cytc, this encompasses the localization of the proteins, the location of the CCM machinery and last but not least the pathways of electron transport from the OCM to the inner compartment. Similar questions arise for the growing number of known double-membraned Archaea including the tiny *Parvarchaeota* of the ARMAN group (Comolli et al., 2009), the *Methanoplasmatales* (methanogens of the *Thermoplasmata* phylum; Dridi et al., 2012; Paul et al., 2012) and the uncultured SM1 euryarchaeota from a newly defined order *Candidatus* “Altiarchaeales” (Figure 1; Probst et al., 2014a,c). The distribution of proteins between the compartments and electron transfer is also unknown in those species. *Ignicoccus spp*. however, are the only double-membraned Archaea with cytochrome *c*. Immuno-labeling had shown that the octaheme MCCs Igni_0955 and Igni_1359 are localized at both membranes (and eventually also at vesicles in the intermembrane compartment (IMC); Figure 5; Naß et al., 2014).

The organisms of the bacterial phylum *Planctomycetes* display ostensibly similar cell morphologies and the question is whether that is comparable to the double-membraned Archaea and whether we can make deductions for protein distribution and electron pathways from these bacteria. *Planctomycetes* species are known to have an inner and outer membrane encompassing a “paryphoplasm” in addition to a protein S-layer (Lindsay et al., 2001; van Teeseling et al., 2014). The paryphoplasm was defined as a structural description of “a unique, peripheral ribosome-free region of cytoplasm” in order to distinguish it from the “riboplasm,” the central compartment containing ribosomes and the nucleoid surrounded by an IM (Lindsay et al., 2001). There is also an ongoing discussion whether or not their membrane organization “is not different from, but an extension of, the “classical” Gram-negative bacterial membrane system” (Santarella-Mellwig et al., 2013; Sagulenko et al., 2014; Jeske et al., in press; van Teeseling et al., in press). Even more complex are Anammox bacteria like *Ca. “*Kuenenia stuttgartiensis,” also belonging to the *Planctomycetes* and also with an S-layer (Figure 5; van Teeseling et al., 2014). The cells have an additional cellular compartment, the anammoxosome within the riboplasm, which contains the proteins required for anaerobic ammonium oxidation including numerous cytochromes *c* like hydroxylamine and hydrazine oxidoreductases. This compartment is the place of energy conversion; the anammoxosome membrane comprises a ph gradient across its ATP synthase-containing membrane with the positive (p) side inside (van Niftrik et al., 2008a, 2010; van der Star et al., 2010; Neumann et al., 2014). Therefore, it is reasonable to assume that maturation of the Cytc (using the system II) takes place in the anammoxosome and that the apoproteins are transported inside. The localization of Cytc is unknown in non-Anammox planctomycetes.

*I. hospitalis* differs in several aspects from the planctomycetes: it does not have an S-layer or a morphologically defined nucleoid and of course nothing equivalent to the anammoxosome. Also, the IMC is very lightly contrasted in electron microscopy pictures suggesting a low concentration of biomolecules. The same seems to be true for the *Methanoplasmatales* (Figure 1; Dridi et al., 2012). In contrast, the paryphoplasm of the planctomycetes usually is much darker in electron microscopy (“electron-dense”) than the IMC (Figure 5; Lindsay et al., 2001). And third, the *I. hospitalis* ATP synthase and a heterologous hydrogenase/sulfur reductase complex are localized in the OCM (Küper et al., 2010). From this, we have to assume that the P-side is outside of the OCM. Maturation of the Cytc at the OCM however would require a transfer of the apoprotein and the heme moiety across two membrane systems (Figure 5). The latter cannot be excluded, however the mature Cytc would have to go back in to reach the IM, where they were found as well (Figure 5). A more easy explanation would be to assume that the apoprotein is transferred co-translationally across the IM via the sec pathway and that maturation would occur prior to further transport. This conclusion however would imply that the maturation takes place in the IMC but at the negative side of the cytoplasmic membrane unless there is an additional proton gradient across the IM. None of that is at present resolved (Huber et al., 2012).

A different question is about the function of the Cytc in *I. hospitalis*. We have proposed that the membrane-bound tetraheme Cytc Igni_0530 might be part of the sulfur reductase, however this is still hypothetical (Naß et al., 2014). Likewise hypothetical is the hypothesis that the Cytc might act as electron relay from the OCM-bound hydrogenase to oxidoreductases in the cytoplasm. We had measured reduction of Igni_0955 (and to a lesser extent Igni_1359) by the native hydrogenase supporting this assumption but it will have to be confirmed independently. At present we would disregard ferredoxins as electron transfer proteins because there are no ferredoxins with twin arginine signal peptides encoded in the *I. hospitalis* genome, which would be required for membrane transport of iron-sulfur proteins. The same observation was made for ferredoxins of *Ca*. “K. stuttgartiensis,” which lead to the—tentative—placement of the ferredoxins in the inner compartment or in the riboplasm, respectively, in the schematic drawings of Figure 5. We also did not find quinones by solvent extraction (Naß et al., 2014). Therefore, the abundantly available Cytc are good candidates for electron transfer from the OCM to the inner compartment in *I. hospitalis*.

We can conclude about the comparison of *I. hospitalis* to the Anammox planctomycetales that the annamoxosomes of those bacteria are distinctly different structures and that the pathways of electron flow and the localization of Cytc is fundamentally different. Unfortunately, we do not know the localization of the respiratory chain(s) in the non-anammox planctomycetes, but they seem to be a system better comparable to the situation in *I. hospitalis* especially regarding Cytc distribution and electron flow.

### Conflict of interest statement

The authors declare that the research was conducted in the absence of any commercial or financial relationships that could be construed as a potential conflict of interest.

## Supplementary material

Table S1 **Kletzin_cytochromes_Archaea_Table_S1.xlsx**. Microsoft Excel file with complete dataset of all archaeal CxxCH motif-containing proteins identified in this study.

Table S2 **Kletzin_cytochromes_Archaea_Table_S2.xlsx**. Microsoft Excel file with summary of sequence clusters with their respective structure prediction results and added remarks.

Igni_0759_pdb.zip **Zipped PDB coordinates of teh Igni_0759 3D model**.

Archaea_Cytc.zip **Compressed file with the sequence clusters aligned in FASTA format**. Cluster 17 is provided in 2 separate files: file “cluster17a.fasta” contains those cluster 17 proteins from Candidatus “Methanoperedens nitroreducens,” which are characterized by a conserved C-terminus.

## References

[B1] Allen J. W. (2011). Cytochrome *c* biogenesis in mitochondria–Systems III and V. FEBS J. 278, 4198–4216. 10.1111/j.1742-4658.2011.08231.x 21736702

[B2] Allen J. W. Harvat E. M. Stevens J. M. Ferguson S. J. (2006). A variant System I for cytochrome *c* biogenesis in Archaea and some Bacteria has a novel CcmE and no CcmH. FEBS Lett. 580, 4827–4834. 10.1016/j.febslet.2006.07.073 16920107

[B3] Anderson I. Risso C. Holmes D. Lucas S. Copeland A. Lapidus A.. (2011). Complete genome sequence of *Ferroglobus placidus* AEDII12DO. Stand. Genomic Sci. 5, 50–60. 10.4056/sigs.2225018 22180810 PMC3236036

[B4] Bandeiras T. M. Refojo P. N. Todorovic S. Murgida D. H. Hildebrandt P. Bauer C.. (2009). The cytochrome *ba* complex from the thermoacidophilic crenarchaeote *Acidianus ambivalens* is an analog of *bc*(1) complexes. Biochim. Biophys. Acta 1787, 37–45. 10.1016/j.bbabio.2008.09.009 18930705

[B5] Baumeister W. Lembcke G. (1992). Structural features of archaebacterial cell envelopes. J. Bioenerg. Biomembr. 24, 567–575. 10.1007/BF00762349 1459988

[B6] Baumeister W. Wildhaber W. Phipps B. M. (1989). Principles of organization in eubacterial and archaebacterial surface proteins. Can. J. Microbiol. 35, 215–227. 10.1139/m89-034 2497940

[B7] Beal E. J. House C. H. Orphan V. J. (2009). Manganese- and iron-dependent marine methane oxidation. Science 325, 184–187. 10.1126/science.1169984 19589998

[B8] Bendtsen J. D. Nielsen H. Widdick D. Palmer T. Brunak S. (2005). Prediction of twin-arginine signal peptides. BMC Bioinformatics 6:167. 10.1186/1471-2105-6-167 15992409 PMC1182353

[B9] Bertini I. Cavallaro G. Rosato A. (2006). Cytochrome *c*: occurrence and functions. Chem. Rev. 106, 90–115. 10.1021/cr050241v 16402772

[B10] Boesen T. Nielsen L. P. (2013). Molecular dissection of bacterial nanowires. MBio 4, e00270–e00213. 10.1128/mBio.00270-13 23653449 PMC3663193

[B11] Burghardt T. Nather D. J. Junglas B. Huber H. Rachel R. (2007). The dominating outer membrane protein of the hyperthermophilic Archaeum *Ignicoccus hospitalis:* a novel pore-forming complex. Mol. Microbiol. 63, 166–176. 10.1111/j.1365-2958.2006.05509.x 17163971

[B12] Cheah K. S. (1970). Properties of the membrane-bound respiratory chain system of *Halobacterium salinarium*. Biochim. Biophys. Acta 216, 43–53. 10.1016/0005-2728(70)90157-X 4993244

[B13] Comolli L. R. Baker B. J. Downing K. H. Siegerist C. E. Banfield J. F. (2009). Three-dimensional analysis of the structure and ecology of a novel, ultra-small archaeon. ISME J. 3, 159–167. 10.1038/ismej.2008.99 18946497

[B14] Delano W. L. (2002). The PyMOL Molecular Graphics System. San Carlos, CA: DeLano Scientific.

[B15] de Vitry C. (2011). Cytochrome *c* maturation system on the negative side of bioenergetic membranes: CCB or system IV. FEBS J. 278, 4189–4197. 10.1111/j.1742-4658.2011.08373.x 21955699

[B16] Dirmeier R. Keller M. Frey G. Huber H. Stetter K. O. (1998). Purification and properties of an extremely thermostable membrane-bound sulfur-reducing complex from the hyperthermophilic *Pyrodictium abyssi*. Eur. J. Biochem. 252, 486–491. 10.1046/j.1432-1327.1998.2520486.x 9546664

[B17] Dridi B. Fardeau M. L. Ollivier B. Raoult D. Drancourt M. (2012). *Methanomassiliicoccus luminyensis* gen. nov., sp. nov., a methanogenic archaeon isolated from human faeces. Int. J. Syst. Evol. Microbiol. 62, 1902–1907. 10.1099/ijs.0.033712-0 22859731

[B18] Feinberg L. F. Holden J. F. (2006). Characterization of dissimilatory Fe(III) versus NO3- reduction in the hyperthermophilic archaeon *Pyrobaculum aerophilum*. J. Bacteriol. 188, 525–531. 10.1128/JB.188.2.525-531.2006 16385043 PMC1347303

[B19] Feinberg L. F. Srikanth R. Vachet R. W. Holden J. F. (2008). Constraints on anaerobic respiration in the hyperthermophilic Archaea *Pyrobaculum islandicum* and *Pyrobaculum aerophilum*. Appl. Environ. Microbiol. 74, 396–402. 10.1128/AEM.02033-07 18039820 PMC2223247

[B20] Forterre P. (2013). The common ancestor of archaea and eukarya was not an archaeon. Archaea 2013:372396. 10.1155/2013/372396 24348094 PMC3855935

[B21] Gorby Y. A. Yanina S. Mclean J. S. Rosso K. M. Moyles D. Dohnalkova A.. (2006). Electrically conductive bacterial nanowires produced by *Shewanella oneidensis* strain MR-1 and other microorganisms. Proc. Natl. Acad. Sci. U.S.A. 103, 11358–11363. 10.1073/pnas.0604517103 16849424 PMC1544091

[B22] Guy L. Ettema T. J. (2011). The archaeal ‘TACK’ superphylum and the origin of eukaryotes. Trends Microbiol. 19, 580–587. 10.1016/j.tim.2011.09.002 22018741

[B23] Hafenbradl D. Keller M. Dirmeier R. Rachel R. Rossnagel P. Burggraf S.. (1996). *Ferroglobus placidus* gen. nov., sp. nov., A novel hyperthermophilic archaeum that oxidizes Fe^2+^ at neutral pH under anoxic conditions. Arch. Microbiol. 166, 308–314. 10.1007/s002030050388 8929276

[B24] Haroon M. F. Hu S. Shi Y. Imelfort M. Keller J. Hugenholtz P.. (2013). Anaerobic oxidation of methane coupled to nitrate reduction in a novel archaeal lineage. Nature 500, 567–570. 10.1038/nature12375 23892779

[B25] Heiden S. Hedderich R. Setzke E. Thauer R. K. (1994). Purification of a two-subunit cytochrome-*b*-containing heterodisulfide reductase from methanol-grown *Methanosarcina barkeri*. Eur. J. Biochem. 221, 855–861. 10.1111/j.1432-1033.1994.tb18800.x 8174566

[B26] Hirokawa T. Boon-Chieng S. Mitaku S. (1998). SOSUI: classification and secondary structure prediction system for membrane proteins. Bioinformatics 14, 378–379. 10.1093/bioinformatics/14.4.378 9632836

[B27] Huber H. Küper U. Daxer S. Rachel R. (2012). The unusual cell biology of the hyperthermophilic Crenarchaeon *Ignicoccus hospitalis*. Antonie Van Leeuwenhoek 102, 203–219. 10.1007/s10482-012-9748-5 22653377

[B28] Jeske O. Schuffler M. Schumann P. Schneider A. Boedeker C. Jogler M.. (in press). Planctomycetes do possess a peptidoglycan cell wall. Nat. Comm. 25964217 10.1038/ncomms8116 PMC4432640

[B29] Jung M. Y. Well R. Min D. Giesemann A. Park S. J. Kim J. G.. (2014). Isotopic signatures of N2O produced by ammonia-oxidizing archaea from soils. ISME J. 8, 1115–1125. 10.1038/ismej.2013.205 24225887 PMC3996685

[B30] Jussofie A. Gottschalk G. (1986). Further-studies on the distribution of cytochromes in Methanogenic bacteria. FEMS Microbiol. Lett. 37, 15–18 10.1111/j.1574-6968.1986.tb01758.x

[B31] Kabashima Y. Sakamoto J. (2011). Purification and biochemical properties of a cytochrome *bc* complex from the aerobic hyperthermophilic archaeon *Aeropyrum pernix*. BMC Microbiol. 11:52. 10.1186/1471-2180-11-52 21396131 PMC3062577

[B32] Kamlage B. Blaut M. (1992). Characterization of cytochromes from *Methanosarcina* strain Gö1 and their involvement in electron transport during growth on methanol. J. Bacteriol. 174, 3921–3927. 1597414 10.1128/jb.174.12.3921-3927.1992 PMC206100

[B33] Kappler U. Maher M. J. (2013). The bacterial SoxAX cytochromes. Cell. Mol. Life Sci. 70, 977–992. 10.1007/s00018-012-1098-y 22907414 PMC11113948

[B34] Kartal B. Maalcke W. J. de Almeida N. M. Cirpus I. Gloerich J. Geerts W.. (2011). Molecular mechanism of anaerobic ammonium oxidation. Nature 479, 127–130. 10.1038/nature10453 21964329

[B35] Katoh K. Standley D. M. (2014). MAFFT: iterative refinement and additional methods. Methods Mol. Biol. 1079, 131–146. 10.1007/978-1-62703-646-7_8 24170399

[B36] Kelley L. A. Sternberg M. J. (2009). Protein structure prediction on the web: a case study using the Phyre server. Nat. Protoc. 4, 363–371. 10.1038/nprot.2009.2 19247286

[B37] Kern M. Klotz M. G. Simon J. (2011). The *Wolinella succinogenes mcc* gene cluster encodes an unconventional respiratory sulphite reduction system. Mol. Microbiol. 82, 1515–1530. 10.1111/j.1365-2958.2011.07906.x 22040142

[B38] Klingl A. (2014). S-layer and cytoplasmic membrane - exceptions from the typical archaeal cell wall with a focus on double membranes. Front. Microbiol. 5:624. 10.3389/fmicb.2014.00624 25505452 PMC4243693

[B39] König H. Rachel R. Claus H. (2007). Proteinaceous surface layers of Archaea: ultrastructure and biochemistry, in Archaea. Molecular and Cellular Biology, ed Cavicchioli R. (Washington, DC: ASM Press), 315–340.

[B40] Krogh A. Larsson B. Von Heijne G. Sonnhammer E. L. (2001). Predicting transmembrane protein topology with a hidden Markov model: application to complete genomes. J. Mol. Biol. 305, 567–580. 10.1006/jmbi.2000.4315 11152613

[B41] Kuhn W. Fiebig K. Hippe H. Mah R. A. Huser B. A. Gottschalk G. (1983). Distribution of cytochromes in Methanogenic bacteria. FEMS Microbiol. Lett. 20, 407–410 10.1016/0378-1097(83)90105-2

[B42] Kunkel A. Vaupel M. Heim S. Thauer R. K. Hedderich R. (1997). Heterodisulfide reductase from methanol-grown cells of *Methanosarcina barkeri* is not a flavoenzyme. Eur. J. Biochem. 244, 226–234. 10.1111/j.1432-1033.1997.00226.x 9063468

[B43] Küper U. Meyer C. Müller V. Rachel R. Huber H. (2010). Energized outer membrane and spatial separation of metabolic processes in the hyperthermophilic Archaeon *Ignicoccus hospitalis*. Proc. Natl. Acad. Sci. U.S.A. 107, 3152–3156. 10.1073/pnas.0911711107 20133662 PMC2840320

[B44] Lanyi J. K. (1968). Studies of the electron transport chain of extremely halophilic bacteria. I. Spectrophotometric identification of the cytochromes of *Halobacterium cutirubrum*. Arch. Biochem. Biophys. 128, 716–724. 10.1016/0003-9861(68)90080-5 5704305

[B45] Lindsay M. R. Webb R. I. Strous M. Jetten M. S. Butler M. K. Forde R. J.. (2001). Cell compartmentalisation in planctomycetes: novel types of structural organisation for the bacterial cell. Arch. Microbiol. 175, 413–429. 10.1007/s002030100280 11491082

[B46] Lockwood C. W. Clarke T. A. Butt J. N. Hemmings A. M. Richardson D. J. (2011). Characterization of the active site and calcium binding in cytochrome *c* nitrite reductases. Biochem. Soc. Trans. 39, 1871–1875. 10.1042/BST20110731 22103542

[B47] Mardanov A. V. Gumerov V. M. Slobodkina G. B. Beletsky A. V. Bonch-Osmolovskaya E. A. Ravin N. V.. (2012). Complete genome sequence of strain 1860, a crenarchaeon of the genus *Pyrobaculum* able to grow with various electron acceptors. J. Bacteriol. 194, 727–728. 10.1128/JB.06465-11 22247528 PMC3264072

[B48] Matias V. R. Beveridge T. J. (2005). Cryo-electron microscopy reveals native polymeric cell wall structure in Bacillus subtilis 168 and the existence of a periplasmic space. Mol. Microbiol. 56, 240–251. 10.1111/j.1365-2958.2005.04535.x 15773993

[B49] Mayer F. Küper U. Meyer C. Daxer S. Müller V. Rachel R.. (2012). AMP-forming acetyl coenzyme A synthetase in the outermost membrane of the hyperthermophilic crenarchaeon *Ignicoccus hospitalis*. J. Bacteriol. 194, 1572–1581. 10.1128/JB.06130-11 22247508 PMC3294865

[B50] Mayer F. Müller V. (2014). Adaptations of anaerobic archaea to life under extreme energy limitation. FEMS Microbiol. Rev. 38, 449–472. 10.1111/1574-6976.12043 24118021

[B51] Meyer C. Heimerl T. Wirth R. Klingl A. Rachel R. (2014). The Iho670 fibers of *Ignicoccus hospitalis* are anchored in the cell by a spherical structure located beneath the inner membrane. J. Bacteriol. 196, 3807–3815. 10.1128/JB.01861-14 25157085 PMC4248799

[B52] Meyerdierks A. Kube M. Kostadinov I. Teeling H. Glockner F. O. Reinhardt R.. (2010). Metagenome and mRNA expression analyses of anaerobic methanotrophic archaea of the ANME-1 group. Environ. Microbiol. 12, 422–439. 10.1111/j.1462-2920.2009.02083.x 19878267

[B53] Moissl C. Rachel R. Briegel A. Engelhardt H. Huber R. (2005). The unique structure of archaeal ‘hami’, highly complex cell appendages with nano-grappling hooks. Mol. Microbiol. 56, 361–370. 10.1111/j.1365-2958.2005.04294.x 15813730

[B54] Monteiro M. Seneca J. Magalhaes C. (2014). The history of aerobic ammonia oxidizers: from the first discoveries to today. J. Microbiol. 52, 537–547. 10.1007/s12275-014-4114-0 24972807

[B55] Mooser D. Maneg O. Macmillan F. Malatesta F. Soulimane T. Ludwig B. (2006). The menaquinol-oxidizing cytochrome *bc* complex from *Thermus thermophilus*: protein domains and subunits. Biochim. Biophys. Acta 1757, 1084–1095. 10.1016/j.bbabio.2006.05.033 16908008

[B56] Naß B. Pöll U. Langer J. D. Kreuter L. Küper U. Flechsler J.. (2014). Three multihaem cytochromes *c* from the hyperthermophilic archaeon *Ignicoccus hospitalis*: purification, properties and localization. Microbiology 160, 1278–1289. 10.1099/mic.0.077792-0 24705227

[B57] Nelson-Sathi S. Dagan T. Landan G. Janssen A. Steel M. Mcinerney J. O.. (2012). Acquisition of 1000 eubacterial genes physiologically transformed a methanogen at the origin of Haloarchaea. Proc. Natl. Acad. Sci. U.S.A. 109, 20537–20542. 10.1073/pnas.1209119109 23184964 PMC3528564

[B58] Nelson-Sathi S. Sousa F. L. Roettger M. Lozada-Chavez N. Thiergart T. Janssen A.. (2015). Origins of major archaeal clades correspond to gene acquisitions from bacteria. Nature 517, 77–80. 10.1038/nature13805 25317564 PMC4285555

[B59] Neumann S. Wessels H. J. Rijpstra W. I. Sinninghe Damste J. S. Kartal B. Jetten M. S.. (2014). Isolation and characterization of a prokaryotic cell organelle from the anammox bacterium *Kuenenia stuttgartiensis*. Mol. Microbiol. 94, 794–802. 10.1111/mmi.12816 25287816

[B60] Noor M. R. Soulimane T. (2013). Structure of *caa*(3) cytochrome *c* oxidase–a nature-made enzyme-substrate complex. Biol. Chem. 394, 579–591. 10.1515/hsz-2012-0343 23399637

[B61] Nunoura T. Sako Y. Wakagi T. Uchida A. (2003). Regulation of the aerobic respiratory chain in the facultatively aerobic and hyperthermophilic archaeon *Pyrobaculum oguniense*. Microbiology 149, 673–688. 10.1099/mic.0.26000-0 12634336

[B62] Offre P. Spang A. Schleper C. (2013). Archaea in biogeochemical cycles. Annu. Rev. Microbiol. 67, 437–457. 10.1146/annurev-micro-092412-155614 23808334

[B63] Paul K. Nonoh J. O. Mikulski L. Brune A. (2012). “*Methanoplasmatales,” Thermoplasmatales*-related archaea in termite guts and other environments, are the seventh order of methanogens. Appl. Environ. Microbiol. 78, 8245–8253. 10.1128/AEM.02193-12 23001661 PMC3497382

[B64] Perras A. K. Wanner G. Klingl A. Mora M. Auerbach A. K. Heinz V.. (2014). Grappling archaea: ultrastructural analyses of an uncultivated, cold-loving archaeon, and its biofilm. Front. Microbiol. 5:397. 10.3389/fmicb.2014.00397 25140167 PMC4122167

[B65] Petersen T. N. Brunak S. von Heijne G. Nielsen H. (2011). SignalP 4.0: discriminating signal peptides from transmembrane regions. Nat. Methods 8, 785–786. 10.1038/nmeth.1701 21959131

[B66] Petitjean C. Deschamps P. Lopez-Garcia P. Moreira D. (2014). Rooting the domain archaea by phylogenomic analysis supports the foundation of the new kingdom *Proteoarchaeota*. Genome Biol. Evol. 7, 191–204. 10.1093/gbe/evu274 25527841 PMC4316627

[B67] Pettersen E. F. Goddard T. D. Huang C. C. Couch G. S. Greenblatt D. M. Meng E. C.. (2004). UCSF Chimera–a visualization system for exploratory research and analysis. J. Comput. Chem. 25, 1605–1612. 10.1002/jcc.20084 15264254

[B68] Pihl T. D. Black L. K. Schulman B. A. Maier R. J. (1992). Hydrogen-oxidizing electron transport components in the hyperthermophilic archaebacterium *Pyrodictium brockii*. J. Bacteriol. 174, 137–143. 1309514 10.1128/jb.174.1.137-143.1992 PMC205687

[B69] Probst A. J. Birarda G. Holman H. Y. Desantis T. Z. Wanner G. Andersen G. L.. (2014a). Coupling genetic and chemical microbiome profiling reveals heterogeneity of archaeome and bacteriome in subsurface biofilms that are dominated by the same archaeal species. PLoS ONE 9:e99801. 10.1371/journal.pone.0099801 24971452 PMC4074051

[B70] Probst A. J. Weinmaier T. Raymann K. Perras A. Emerson J. B. Rattei T.. (2014c). Biology of a widespread uncultivated archaeon that contributes to carbon fixation in the subsurface. Nat. Commun. 5, 5497. 10.1038/ncomms6497 25425419

[B71] Protze J. Müller F. Lauber K. Nass B. Mentele R. Lottspeich F.. (2011). An extracellular Tetrathionate Hydrolase from the Thermoacidophilic Archaeon *Acidianus ambivalens* with an activity optimum at pH 1. Front. Microbiol. 2:68. 10.3389/fmicb.2011.00068 21747790 PMC3128947

[B72] Rachel R. Meyer C. Klingl A. Gurster S. Heimerl T. Wasserburger N.. (2010). Analysis of the ultrastructure of archaea by electron microscopy. Methods Cell Biol. 96, 47–69. 10.1016/S0091-679X(10)96003-2 20869518

[B73] Richter K. Schicklberger M. Gescher J. (2012). Dissimilatory reduction of extracellular electron acceptors in anaerobic respiration. Appl. Environ. Microbiol. 78, 913–921. 10.1128/AEM.06803-11 22179232 PMC3273014

[B74] Romão C. V. Archer M. Lobo S. A. Louro R. O. Pereira I. A. C. Saraiva L. M. (2012). Diversity of heme proteins in sulfate reducing bacteria, in Handbook of Porphyrin Science, eds Kadish K. M. Smith K. M. Guilard R. (Singapore: World Scientific Publishing Co), 139–229.

[B75] Roy A. Kucukural A. Zhang Y. (2010). I-TASSER: a unified platform for automated protein structure and function prediction. Nat. Protoc. 5, 725–738. 10.1038/nprot.2010.5 20360767 PMC2849174

[B76] Sagulenko E. Morgan G. P. Webb R. I. Yee B. Lee K. C. Fuerst J. A. (2014). Structural studies of planctomycete Gemmata obscuriglobus support cell compartmentalisation in a bacterium. PLoS ONE 9:e91344. 10.1371/journal.pone.0091344 24632833 PMC3954628

[B77] Santarella-Mellwig R. Pruggnaller S. Roos N. Mattaj I. W. Devos D. P. (2013). Three-dimensional reconstruction of bacteria with a complex endomembrane system. PLoS Biol. 11:e1001565. 10.1371/journal.pbio.1001565 23700385 PMC3660258

[B78] Schäfer G. (2004). Respiratory chains in Archaea: from minimal systems to supercomplexes, in Respiration in Archaea and Bacteria. Diversity of Prokaryotic Respiratory Systems, ed Zannoni D. (Dordrecht: Springer), 1–33.

[B79] Schäfer G. Engelhard M. Müller V. (1999). Bioenergetics of the Archaea. Microbiol. Mol. Biol. Rev. 63, 570–620. 10477309 10.1128/mmbr.63.3.570-620.1999 PMC103747

[B80] Scharf B. Wittenberg R. Engelhard M. (1997). Electron transfer proteins from the haloalkaliphilic archaeon *Natronobacterium pharaonis*: possible components of the respiratory chain include cytochrome *bc* and a terminal oxidase cytochrome *ba*3. Biochemistry 36, 4471–4479. 10.1021/bi962312d 9109654

[B81] Seiler M. Mehrle A. Poustka A. Wiemann S. (2006). The 3of5 web application for complex and comprehensive pattern matching in protein sequences. BMC Bioinformatics 7:144. 10.1186/1471-2105-7-144 16542452 PMC1523217

[B82] Sharma S. Cavallaro G. Rosato A. (2010). A systematic investigation of multiheme *c*-type cytochromes in prokaryotes. J. Biol. Inorg. Chem. 15, 559–571. 10.1007/s00775-010-0623-4 20084531

[B83] Simon J. Hederstedt L. (2011). Composition and function of cytochrome *c* biogenesis System II. FEBS J. 278, 4179–4188. 10.1111/j.1742-4658.2011.08374.x 21955752

[B84] Simon J. Kern M. Hermann B. Einsle O. Butt J. N. (2011). Physiological function and catalytic versatility of bacterial multihaem cytochromes *c* involved in nitrogen and sulfur cycling. Biochem. Soc. Trans. 39, 1864–1870. 10.1042/BST20110713 22103541

[B85] Smith J. Aklujkar M. Risso C. Leang C. Giloteaux L. Holmes D. (2015). Mechanisms involved in Fe(III) respiration by the hyperthermophilic archaeon *Ferroglobus placidus*. Appl. Environ. Microbiol. 81, 2735–2744. 10.1128/AEM.04038-14 25662973 PMC4375341

[B86] Sreeramulu K. (2003). Purification and partial characterization of a cytochrome *c_552_* from *Halobacterium salinarum*. Ind. J. Biochem. Biophys. 40, 274–277. 22900320

[B87] Sreeramulu K. Schmidt C. L. Schäfer G. Anemüller S. (1998). Studies of the electron transport chain of the euryarcheon *Halobacterium salinarum*: indications for a type II NADH dehydrogenase and a complex III analog. J. Bioenerg. Biomembr. 30, 443–453. 10.1023/A:1020538129400 9932647

[B88] Stevens J. M. Mavridou D. A. Hamer R. Kritsiligkou P. Goddard A. D. Ferguson S. J. (2011). Cytochrome c biogenesis system I. FEBS J. 278, 4170–4178. 10.1111/j.1742-4658.2011.08376.x 21958041 PMC3601427

[B89] Stieglmeier M. Klingl A. Alves R. J. Rittmann S. K. Melcher M. Leisch N.. (2014). Nitrososphaera viennensis gen. nov., sp. nov., an aerobic and mesophilic, ammonia-oxidizing archaeon from soil and a member of the archaeal phylum Thaumarchaeota. Int. J. Syst. Evol. Microbiol. 64, 2738–2752. 10.1099/ijs.0.063172-0 24907263 PMC4129164

[B90] Tan K. Duquette M. Liu J. H. Lawler J. Wang J. H. (2008). The crystal structure of the heparin-binding reelin-N domain of f-spondin. J. Mol. Biol. 381, 1213–1223. 10.1016/j.jmb.2008.06.045 18602404 PMC2561254

[B91] Tanaka M. Ogawa N. Ihara K. Sugiyama Y. Mukohata Y. (2002). Cytochrome *aa*(3) in *Haloferax volcanii*. J. Bacteriol. 184, 840–845. 10.1128/JB.184.3.840-845.2002 11790755 PMC139504

[B92] Thauer R. K. Kaster A. K. Seedorf H. Buckel W. Hedderich R. (2008). Methanogenic archaea: ecologically relevant differences in energy conservation. Nat. Rev. Microbiol. 6, 579–591. 10.1038/nrmicro1931 18587410

[B93] van der Star W. R. Dijkema C. de Waard P. Picioreanu C. Strous M. Van Loosdrecht M. C. (2010). An intracellular pH gradient in the anammox bacterium *Kuenenia stuttgartiensis* as evaluated by 31P NMR. Appl. Microbiol. Biotechnol. 86, 311–317. 10.1007/s00253-009-2309-9 19862513 PMC2822221

[B94] van Niftrik L. Geerts W. J. van Donselaar E. G. Humbel B. M. Webb R. I. Fuerst J. A.. (2008a). Linking ultrastructure and function in four genera of anaerobic ammonium-oxidizing bacteria: cell plan, glycogen storage, and localization of cytochrome *c* proteins. J. Bacteriol. 190, 708–717. 10.1128/JB.01449-07 17993524 PMC2223682

[B95] van Niftrik L. Geerts W. J. van Donselaar E. G. Humbel B. M. Yakushevska A. Verkleij A. J.. (2008b). Combined structural and chemical analysis of the anammoxosome: a membrane-bounded intracytoplasmic compartment in anammox bacteria. J. Struct. Biol. 161, 401–410. 10.1016/j.jsb.2007.05.005 17604181

[B96] van Niftrik L. van Helden M. Kirchen S. van Donselaar E. G. Harhangi H. R. Webb R. I.. (2010). Intracellular localization of membrane-bound ATPases in the compartmentalized anammox bacterium *‘Candidatus Kuenenia stuttgartiensis’*. Mol. Microbiol. 77, 701–715. 10.1111/j.1365-2958.2010.07242.x 20545867 PMC2936114

[B97] van Teeseling M. C. de Almeida N. M. Klingl A. Speth D. R. Op den Camp H. J. Rachel R.. (2014). A new addition to the cell plan of anammox bacteria: “*Candidatus Kuenenia stuttgartiensis”* has a protein surface layer as the outermost layer of the cell. J. Bacteriol. 196, 80–89. 10.1128/JB.00988-13 24142254 PMC3911120

[B98] van Teeseling M. C. F. Mesman R. J. Kuru E. Espaillat A. Cava F. Brun Y. V.. (in press). Anammox Planctomycetes have a peptidoglycan cell wall. Nat. Comm. 25962786 10.1038/ncomms7878 PMC4432595

[B99] van Teeseling M. C. Neumann S. van Niftrik L. (2013). The anammoxosome organelle is crucial for the energy metabolism of anaerobic ammonium oxidizing bacteria. J. Mol. Microbiol. Biotechnol. 23, 104–117. 10.1159/000346547 23615199

[B100] Veith A. Klingl A. Zolghadr B. Lauber K. Mentele R. Lottspeich F.. (2009). *Acidianus, Sulfolobus* and *Metallosphaera* surface layers: structure, composition and gene expression. Mol. Microbiol. 73, 58–72. 10.1111/j.1365-2958.2009.06746.x 19522740

[B101] Verissimo A. F. Daldal F. (2014). Cytochrome *c* biogenesis system I: an intricate process catalyzed by a maturase supercomplex? Biochim. Biophys. Acta 1837, 989–998. 10.1016/j.bbabio.2014.03.003 24631867 PMC4047167

[B102] Wang F. P. Zhang Y. Chen Y. He Y. Qi J. Hinrichs K. U.. (2014). Methanotrophic archaea possessing diverging methane-oxidizing and electron-transporting pathways. ISME J. 8, 1069–1078. 10.1038/ismej.2013.212 24335827 PMC3996691

[B103] Wang M. Tomb J. F. Ferry J. G. (2011). Electron transport in acetate-grown *Methanosarcina acetivorans*. BMC Microbiol. 11:165. 10.1186/1471-2180-11-165 21781343 PMC3160891

[B104] Wankel S. D. Adams M. M. Johnston D. T. Hansel C. M. Joye S. B. Girguis P. R. (2012). Anaerobic methane oxidation in metalliferous hydrothermal sediments: influence on carbon flux and decoupling from sulfate reduction. Environ. Microbiol. 14, 2726–2740. 10.1111/j.1462-2920.2012.02825.x 22827909

[B105] Wu M. L. Van Teeseling M. C. Willems M. J. Van Donselaar E. G. Klingl A. Rachel R.. (2012). Ultrastructure of the denitrifying methanotroph “*Candidatus Methylomirabilis oxyfera,”* a novel polygon-shaped bacterium. J. Bacteriol. 194, 284–291. 10.1128/JB.05816-11 22020652 PMC3256638

